# Association between maternal age and adverse perinatal outcomes in Arba Minch zuria, and Gacho Baba district, southern Ethiopia: a prospective cohort study

**DOI:** 10.1186/s12884-020-03285-0

**Published:** 2020-10-06

**Authors:** Abera Mersha, Gistane Ayele, Tilahun Worku, Zerihun Zerdo, Shitaye Shibiru, Agegnehu Bante, Tamiru Chonka

**Affiliations:** 1 School of Nursing, College of Medicine and Health Sciences, Arba Minch University, Arba Minch, Ethiopia; 2 School of Public Health, College of Medicine and Health Sciences, Arba Minch University, Arba Minch, Ethiopia; 3 Department of Medical Laboratory Sciences, College of Medicine and Health Sciences, Arba Minch University, Arba Minch, Ethiopia

**Keywords:** Advanced maternal age, Perinatal outcomes, Arba Minch

## Abstract

**Background:**

Globally, delayed childbearing to the advanced age is a growing option. It is an emerging public health issue in developing countries. Currently, adverse perinatal outcomes significantly increased. A few studies showed the effect of advanced maternal age on adverse perinatal outcomes. However, most used secondary data or chart reviews, and this increases the risk of biases. Besides, there are limited studies in-country Ethiopia as advanced maternal age steadily increased. Therefore, this study aimed to assess the status of advanced maternal age and its effect on perinatal outcomes in the study setting.

**Methods:**

A community-based prospective cohort study was conducted among 709 study participants from October 15, 2018, to September 30, 2019, in Arba Minch zuria, and Gacho Baba district, southern Ethiopia. The data were collected by a pretested interviewer-administered structured Open Data Kit survey tool and analyzed by SPSS version 25. The log-linear regression model was used to compare perinatal outcomes among women aged 20–34 years and ≥ 35 years. The log-likelihood ratio tested for the goodness of fit. In this study, *P*-value < 0.05 was considered to declare a result as a statistically significant association.

**Results:**

In this study, 209(29.5%) of the women were age group ≥35 years old, and 500(70.5%) were age group from20–34 years old. Stillbirth (β = 0.29, 95%CI: 0.05, 0.52), and neonatal mortality (β = 0.11, 95%CI: 0.01, 0.21) were significantly associated with the advanced maternal age.

**Conclusions:**

Perinatal outcomes such as stillbirth and neonatal mortality were independently associated with advanced maternal age after controlling for possible cofounders. Therefore, different strategies should design for the women who planned to bear child, and information should provide for women who are advanced age or delayed childbearing to alert them.

## Background

### Advanced maternal age, the reason, and its consequences in a global context, and in Africa

Advanced maternal age (AMA) means the age group of 35 years or older [1–3]. Worldwide, delayed childbearing is a growing option [4–6]. The prevalence of pregnant women with AMA was 12.3% in the study conducted in 359 health facilities of 29 countries in Africa, Asia, Latin America, and the Middle East [7]. A study from Israel showed 45.7% of women were AMA, 33.4% in the study from Norway, and 14.8% in the report from Malaysia [8–10].

Belated marriage, a higher level of education, career pursuit, increased life expectancy, contraceptive use, labor market participation, economic uncertainty and value changes were the main reasons for delayed childbearing [11–13]. On the contrary, some studies indicated that multiparous women continuing childbearing to advanced maternal age because of ignorance, low use of contraceptive methods, and remarriage [14, 15]. The socio-economic and technological changes have significantly contributed to increasing the number of women who delay childbearing. This demographic shift speculated as a public health issue, and it becomes challenging for both patients and clinicians. Delaying pregnancy too advanced age increased the risk of adverse perinatal outcomes [4, 6, 10, 16–19].

Women with AMA revealed a significantly increased risk of prematurity and unfavorable perinatal outcomes [20]. Evidence from different studies indicated that AMA significantly increased the risk of prematurity, fetal mortality, early neonatal mortality, perinatal mortality, low birth weight, and birth asphyxia [1, 2, 5, 7–9, 21–23]. A result of a study conducted in Nepal showed the older women had a significantly higher incidence of perinatal death (7.7% vs. 0%) [24]. Similarly, a study conducted in South Africa reported that older women had an increased risk of perinatal death as compared to the younger ones [25].

### Impact of advanced maternal age in Ethiopia

Findings from Ethiopia revealed that AMA was increasingly associated with adverse perinatal outcomes like prematurity, low birth weight, perinatal death, stillbirth, and low fifth-minute Apgar score [26, 27].

### The rationale of the study

Thus, studies on the effect of advanced maternal age on perinatal outcomes are very important for policymakers and program evaluators to improve perinatal health. Some studies conducted in different settings [1, 2, 5, 7–9, 21–23]. Nevertheless, most were conducted retrospectively and depend on charts or record reviews, which is more prone to biases. Besides, there are limited studies in-country Ethiopia as AMA steadily increasing. Therefore, this study aimed to assess the status of advanced maternal age and its effect on perinatal outcomes in the study setting.

### Hypothesis

This study hypothesized that advanced maternal age increases the risk of adverse perinatal outcomes after controlling possible confounding variables.

## Methods

### Study setting and period

In this study, women’s in Arba Minch zuria, and Gacho Baba district, Arba Minch-Health, and Demographic Surveillance System sites (AM-HDSS), southern Ethiopia involved, from October 15, 2018, to September 30, 2019. Arba Minch-Health and Demographic Surveillance System sites were established in collaboration between Arba Minch University and Ethiopian Public Health Association with the support of the Centers for Disease Control and Prevention (CDC) Ethiopia in 2009 to track demographic changes. The surveillance site included nine kebeles from the 29 kebeles located in Arba Minch zuria, and Gacho Baba district, Gamo zone, southern Ethiopia [28]. Arba Minch is an administrative town in the Gamo zone, located 505 km south of Addis Ababa and 275 km southwest of Hawassa. Based on the 2007 Census conducted by the Central Statistical Agency (CSA), these districts have a total population of 164,529, of whom 82,199 are men and 82,330 women. According to the HDSS report, there is a total population of 74,157 in the surveillance site.

### Study design

A community-based prospective cohort study design was employed to meet study objectives.

### Population

#### Source population

The source population for this study was all women who were pregnant in Arba Minch zuria, and Gacho Baba district, AM-HDSS site, southern Ethiopia.

#### Study population

Those women who were pregnant during the study period (2018–2019) were study population for this study.

#### Inclusion criteria

At enrollment for this study, all women who were pregnant and inhabitants to a minimum of six months in the study area were eligible for this study. The eligibility defined by the pregnancy screening checklist, which was developed by Whiteman et al. [29].

#### Exclusion criteria

During recruitment, all women whose ages less than twenty years old and known to be preexisting illnesses excluded from the study.

### Sample size determination

Epi info7 software Stat Calc used to estimate the sample sizes. For the first objective, a single population proportion was used by considering the following assumptions: *P* = 0.334 from the study conducted in Norway [9], 95% level of confidence, and 5% margin of error used. Based on this, the estimated sample size was 342. A two-sample comparison proportion used to estimate the sample size for the second objective. The assumption was P_1_ (age group 20–34) = 0.207 and P_2_ (age group ≥35) = 0.124 in the study conducted in Malaysia [10], 95%CI, ratio 1:1, and Power = 80% and the sample size estimated by this assumption was 676. The sample size for this study estimated by adding a non-response rate of 10% to the larger sample size. Therefore, the calculated sample size for this study was 744.

### Data collection tool

The data were collected using a pretested interviewer-administered structured Open Data Kit (ODK) survey tool. The tools were developed by reviewing different works of literature. The wealth index assessment questionnaire adapted from the questionnaire used in the Ethiopian Demographic Health Survey (EDHS) 2016 [30]. The household food insecurity level measured with Household Food Insecurity Access Scale (HFIAS), a structured, standardized, and validated tool that developed mainly by Food and Nutrition Technical Assistance (FANTA) [31]. They have three main parts for the questionnaire: Part I (pregnancy screening checklist), Part II (baseline information), and Part III (follow-up survey tool) (Additional file 1).

### Pretest

The tools pretested in the Chencha district, which was out of the study area to verify the appropriateness, and modifications and amendments were taken accordingly before actual data collection.

### Data collection procedures

The well-trained nine data collectors and three field supervisors were prospectively identified perinatal outcomes among pregnant women during the study period. Intensive three days training gave for data collectors and supervisors separately regarding objectives of the study and data collection ways. Data collectors discussed the information about the ODK survey tool and pregnancy screening checklists to identify pregnant women. The data collected in different phases, as this was a community-based prospective follow-up study. In the first phase: all the baseline information about the women obtained and pregnancy status was checked by using a pregnancy-screening checklist. After identified whether women were advanced age or not, and the data collectors have recruited the women into the cohort. In the second phase: the women were followed started from the time pregnancy confirmed up to the immediate postpartum period to identify some of the perinatal outcomes. The follow-up terminated at the end of the neonatal period that the neonates reassessed with a similar fashion in the above mechanism. In the community setting, the data collectors frequently contacted women or any household members, surround health care institutions, and health extension workers during the follow-up period.

### Study variables and measurements

The description and measurements for some of the outcome and explanatory variables were stated in detail below (Table 1).

**Table 1:** Measurements to assess the status of advanced maternal age and their effect on perinatal outcomes in Arba Minch zuria, and Gacho Baba district, southern Ethiopia, 2018/9

Variables	Description	Measurements
**Perinatal outcomes**		
*Gestational age*	A period counted from the Last Normal Menstrual Period (LNMP) if the mother remembered, or based on Ultrasound result during pregnancy.	Neonate’s gestational age categorized as pre-term for less than 37 weeks coded as “1”, a term for 37–42 weeks coded as “2′, and post-term 42+ weeks coded as “3″.
*Size of the neonate*	Birth weight or size of the neonate during delivery.	The size of the neonate categorized as very small coded as “1”, smaller than usual coded as “2”, about average coded as “3”, and larger than usual coded as “4”.
*Stillbirth*	Give birth to a dead fetus after 28 weeks of gestation.	Those conceptuses ended up with stillbirth coded as “1′, and the other coded as “2″.
*Neonatal mortality*	Neonates died within 28 days of birth.	Those neonates died within 28 days by the non-accident case were coded as “1”, not were coded as “2”.
**Exposure variable**		
*Advanced maternal age*	Defined as a pregnant mother aged ≥35 years old [17].	Categorized into two groups, and for the mother aged 20–34 years old was coded as “1” and “2” for ≥35 years.
**Adjusted/confounding and some other variables**		
*Parity*	Number of births that the woman have	The responses categorized into two categories as primi (1st birth order) coded as “1” and multipara (2 or more birth order coded as “2”.
*BMI*	Weight of women in kg per height square	Classified into underweight (< 18.5 kg/m2), normal (18.5–24.9 kg/m2), overweight (25–29.9 kg/m2), obese (30–34.9 kg/m2), and morbidly obese (≥35 kg/m2).
*Wealth index*	The EDHS household assets questions used, and principal component analysis done to rank the categories.	It ranked into three categories, 1st quantile coded as 1, 2nd quantile coded as “2”, and 3rd quantile coded as “3”.
*Distance to the health center*	Approximate distance to the health center on foot which was responded by the respondent	Categorized in to two: “1” = ≤2 h on foot and “2”= > 2 h (BEmOC)
*Distance to the hospital*	Approximate distance to the hospital on foot which was responded by the respondent	Categorized in to two: “1” = ≤2 h on foot and “2”= > 2 h (CEmOC)
*Household food insecurity*	Both physical and economic access to sufficient food to meet their dietary needs for a productive and healthy life	Categorized households into four levels of household food insecurity (access) based on response to nine questions of HFIAS: food secure (1) and mild (2), moderately (3), and severely food insecure (4) [31].
*Districts (woreda)*	It is the third-level administrative divisions of Ethiopia.	
*Kebele (wards)*	Defined as the smallest administrative unit of Ethiopia, and it is a neighborhood or a localized and delimited group of people.	

### Data quality assurance

To ensure quality, experts translated questionnaires into the local language. A standard tool, which was commented by many experts, was used to collect the information. The data collectors and supervisors trained to standardize and ensure consistency of data collection. The principal investigator and supervisors critically checked the data for completeness before uploaded to the ODK cloud server. Multiple imputation techniques used for the missed data that were not more than 20% of the needed information. The inconsistent data excluded from the final analysis. The data coded correctly and categorized to maintain quality.

### Data processing and analysis

The collected data were downloaded from ODK aggregate and exported to SPSS version 25 for analysis. Proportions and summary statistics computed with maternal age. The wealth quintiles determined by the Principal Component Analysis (PCA). A crude and adjusted log-linear regression analysis was done for each outcome variable with maternal age to estimate the beta coefficient (β). The assumptions for log-linear regression checked, and the log-likelihood ratio tested for the goodness of fit. To control the confounding effect, all the variables with *P* ≤ 0.25 in the bivariate analysis included in the final model. The model adjusted for educational and occupational status, parity, wealth index, body mass index (BMI), HFIAS, lifestyle factors, distance to health care institution, and sex of the neonate, antenatal care, postnatal care, and place of delivery. A standard error of > 2 considered as suggestive of the existence of multi co-linearity. In this study, *P* < 0.05, considered to declare a result as a statistically significant association. Then the information, presented in simple frequencies, summary measures, tables, and figures.

## Results

**Fig. 1:**
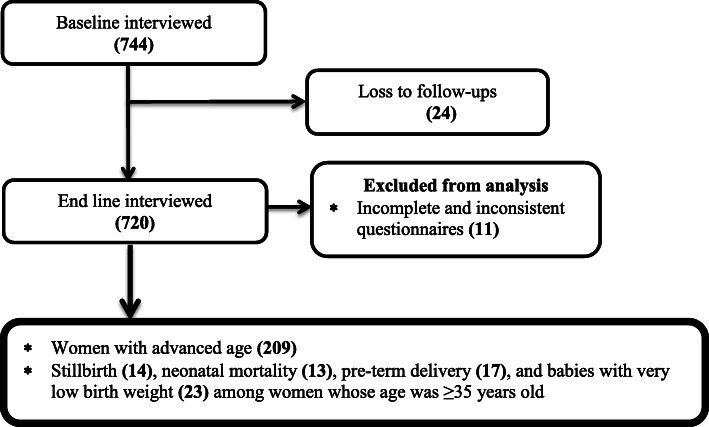
Overall process of the study conducted in Arba Minch zuria, and Gacho Baba district, southern Ethiopia, 2018/9

In this follow-up study, 744 women’s interviewed in the baseline based on the calculated sample size, and 709 completed the follow-up period, which gave the response rate of 95.3% (Fig. 1).

### Socio-demographic and economic characteristics with maternal age

Out of study participants, 500 (70.5%) were age ranged from 20 to 34 years old, and 209(29.5%) were ≥ 35 years old. From women whose age group 20–34 years old, 308(61.6) had no formal education, 143(28.6) had primary, 35(7.0) secondary, and 14(2.8) college and above, and for age group ≥35 years old, 144(68.9) had no formal education, and 47(22.5), 13(6.2), and five (2.4) had primary, secondary, and college and above respectively. Four hundred ninety-six (99.2%) of participants married for the age group 20–34 years and 208(99.5) for age ≥ 35 years (Table 2).

**Table 2:** Socio-demographic and economic characteristics with maternal age for the study conducted in Arba Minch zuria, and Gacho Baba district, southern Ethiopia, 2018/9

Variables	20–34 years	≥ 35 years
**Educational status of the husband**		
No formal education	254 (50.8)	110 (52.6)
Primary(1–8)	171 (34.2)	76 (36.4)
Secondary(9–12)	58 (11.6)	16 (7.7)
College and above	17 (3.4)	7 (3.3)
**Occupation of the mother**		
Housewife	452 (90.4)	194 (92.8)
Other**©**	48 (9.6)	15 (7.2)
**Occupation of the husband**		
Farmer	358 (71.6)	178 (85.2)
Other**±**	142 (28.4)	31 (14.8)
**BMI (kg/m2)**		
Underweight (< 18.5)	35 (7.0)	15 (7.1)
Normal (18.5–24.9)	374 (74.8)	132 (63.2)
Overweight (25–29.9)	91 (18.2)	62 (29.7)
**The average distance from the health post (on foot)**		
≤ 2 h	485 (97.0)	206 (98.6)
> 2 h	15 (3.0)	3 (1.4)
**The average distance from the health center (on foot)**		
≤ 2 h	457 (91.4)	193 (92.3)
> 2 h	43 (8.6)	16 (7.7)
**The average distance from the hospital (on foot)**		
≤ 2 h	131 (26.2)	87 (41.6)
> 2 h	369 (73.8)	122 (58.4)
**Wealth index**		
First quantile	178 (35.6)	59 (28.2)
Second quantile	171 (34.2)	76 (36.4)
Third quantile	151 (30.2)	74 (35.4)

**©** merchant, government employer, daily laborer, student, and farmer, **±** carpenters, Manson, merchant, government employer, daily laborer, private worker, religious leader, student, and driver, and BMI: Body mass index

### Household food insecurity access scale with maternal age

**Fig. 2:**
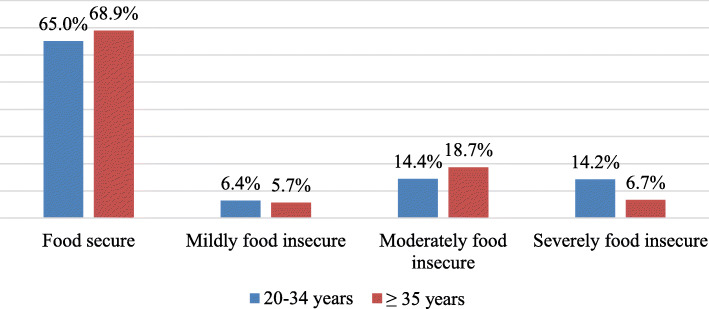
Household food insecurity access scale with maternal age for the study conducted in Arba Minc Zuria, Gacho Baba District, AM-HDSS site, Southern Ethiopia, 2018/9

Of the women whose age was 20–34 years old, 325 (65.0%) and 144 (68.9%) whose age 35 years old or more were food secure (Fig. 2).

### Maternal characteristics with age

Out of study participants, 88(17.6%) of study participants were primipara for age 20–34 years, 16(7.7%) for ≥35 years. Antenatal care was followed by 363(72.6%) of women age 20–34 years and 151(72.2%) for 35 years or more (Table 3).

**Table 3:** Maternal characteristics with age for the study conducted in Arba Minch zuria, and Gacho Baba district, southern Ethiopia, 2018/9

Variables	20–34 years	≥35 years
**ANC visits**		
No visit	137 (27.4)	58 (27.8)
1–3 visits	151 (30.2)	70 (33.5)
Four or more visits	212 (42.4)	81 (38.8)
**Place of delivery**		
Hospital	32 (6.6)	16 (8.7)
Health center	145 (29.8)	32 (17.5)
Health post	69 (14.2)	19 (10.4)
Home	241 (49.4)	116 (63.4)
**Mode of delivery**		
Spontaneous vaginally	429 (88.1)	169 (92.4)
Cesarean section	18 (3.7)	7 (3.8)
Assisted delivery	40 (8.2)	7 (3.8)
**Postnatal care**		
Yes	112 (23.0)	34 (18.6)
No	375 (77.0)	149 (81.4)

*ANC* Antenatal care

### Lifestyle factors of the women with age

Regarding lifestyle factors of the women, 20(4.0%) and 93 (18.6%) had smoking and consumed alcohol-containing beverages for the age group from 20 to 34 years, and 14(6.7%), and 40 (19.1%) for age category 35 years old or more respectively. Thirty-two (6.4%) of women with group 20–34, and 11 (5.3%) for age group ≥35 years old used hashish/shisha/suret, and 36(7.2%) of the women with group 20–34, and 20(9.6%) for age group ≥35 years old consumed local herbs.

### Perinatal characteristics with maternal age

Of the neonates, 228(46.8%) were male for the women aged 20–34 years, and 104(56.8%) for the age group ≥35 years. Ten (2.1%) of the neonates encountered complications during the period for the age group 20–34 years, and four (2.2%) for women aged 35 years or more (Table 4).

**Table 4:** Perinatal characteristics with maternal age for the study conducted in Arba Minch zuria, and Gacho Baba district, southern Ethiopia, 2018/9

Variables	20–34 years	≥35 years
**Stillbirth**		
Yes	11 (2.2)	14 (7.1)
No	487 (97.8)	183 (92.9)
**Gestational age**		
Estimated at term	418 (85.8)	152 (83.1)
Estimated pre-term	21 (4.3)	17 (9.3)
Estimated post-term	48 (9.9)	14 (7.6)
**Size of the neonate**		
Very small	79 (16.2)	23 (12.6)
Smaller than usual	46 (9.4)	11 (6.0)
About average	328 (67.4)	140 (76.5)
Larger than usual	34 (7.0)	9 (4.9)
**The baby referred to another facility**		
Yes	5 (1.0)	2 (1.1)
No	482 (99.0)	181 (98.9)
**Admitted to NICU**		
Yes	7 (1.4)	1 (0.5)
No	480 (98.6)	182 (99.5)
**Neonatal mortality**		
Yes	11 (2.3)	13 (7.1)
No	476 (97.7)	170 (92.9)

*NICU* Neonatal intensive care unit

### Association of maternal age with adverse perinatal outcomes

The adjusted model showed a significant association between maternal age and neonatal mortality and stillbirth. Women with age group ≥35 years old had significantly increased the risk of neonatal mortality and stillbirth as compared to the age group ranged from 20 to 34 years (β =0.11; 95% CI: 0.01, 0.21), and (β =0.29; 95% CI: 0.05, 0.52) respectively (Table 5).

**Table 5:** Association of maternal age to adverse perinatal outcomes for the study conducted in Arba Minch zuria, and Gacho Baba district, southern Ethiopia, 2018/9

Perinatal outcomes	Maternal age ≥ 35 years	
	Crude estimate β	Adjusted estimate ± β
**Stillbirth**	0.30(0.10,0.50)	0.29(0.05,0.52)*
**Pre-term**	0.30(0.07,0.54)	0.11(−0.07,0.28)
**Post-term**	−0.20(−0.42,0.02)	−0.01(−0.19,0.14)
**Estimated small**	−0.02(−0.23,0.19)	0.002(−0.04,0.04)
**Estimated smaller than usual**	−0.10(− 0.37,0.16)	0.007(− 0.03,0.05)
**Estimated larger than usual**	−0.05(− 0.34,0.24)	0.007(− 0.03,0.05)
**Neonatal mortality**	0.29(0.09,0.49)	0.11(0.01,0.21)*

±adjusted for educational status, occupational status, parity, wealth index, BMI, HFIAS, lifestyle factors, distance to health care institutions, sex of the neonate, antenatal care, postnatal care, and place of delivery and *significant at *P* < 0.05

## Discussion

This prospective cohort study aimed to fill research gaps in Ethiopia in assessing the status of advanced maternal age and their effect on the perinatal outcomes. In this finding, 1/3rd of women became pregnant during advanced maternal age. This study reported the association of advanced maternal age to adverse perinatal outcomes, such as stillbirth and neonatal mortality.

In this study, the status of AMA was 29.5%(95%CI: 26.1, 32.8%). This was higher than studies conducted in Israel (2.3 and 14%) [4, 8], Malaysia (14.8%) [10], WHO Multicountry Survey (12.8%) [7], Northeastern Brazil (5.9%) [32], Nepal (4.53%) [24], South Africa (17.5%) [25], and United Kingdom (18.18%) [33]. However, it was lower than studies done in Iran (50.2%) [12], and Norway (33.4%) [9]. This discrepancy may be due to differences in socio-demographic and economic characteristics, technological and health care systems, and socio-cultural factors that lead to delayed childbearing or having pregnant at an advanced age. Typically, as this study conducted in a low-income country that women continue childbearing due to a shortage of contraceptive means and lack of knowledge on how to utilize it.

Finding from this study indicated that the risk of stillbirth significantly increased in AMA. These were congruent with studies conducted in the United Kingdom [33], Washington, United States [34], and in Ethiopia [26, 27]. Correspondingly, neonatal mortality was also associated with AMA in this study. These were in line with studies conducted in Israel [4], South Australia [35], Scotland [36], Brazil [37], and Washington, United States [34]. The reason is that those old blood vessels in the uterus cause uteroplacental insufficiency. In advanced maternal age, the physical ability to bear the child in advanced maternal age may result in adverse perinatal outcomes such as stillbirth. Besides, as maternal age advances high likely give birth to a baby with different complications which unable to adapt extrauterine life and increased risk or more susceptible to infections.

After controlling for confounders, prematurity and post-term delivery were not significantly associated with AMA. This not in line with studies conducted in the United Kingdom [33], South Australia [35], Brazil [16], South Korea [18], Israel [19], and Ethiopia [26]. In this study, the estimated birth weight of babies was not significantly associated with AMA. These contradicted with the studies conducted in Brazil [16] and South Korea [18]. The reason for the discrepancy may be the methodological aspects (source population, sampling, and study participant characteristics) and socio-economic status.

The finding of this study is input for public health. In advanced maternal age, the risk of adverse perinatal outcomes increased significantly. Due to advanced health care delivery systems and technology and socio-economic status, the number of women who delayed childbearing increased from time to time. As such, studies on the effect of advanced maternal age on the adverse perinatal outcomes are vital to strengthen the intervention for women who planned to bear a child. The finding of this study initiates different stakeholders in the health care system to design appropriate strategies and planning for intervention. This study becomes one input for health policymakers and program developers typical regarding perinatal health.

The limitations of this were; some of the medical words were difficult to translate to the local language exactly. Some values are difficult to set cut off points, and based on the maternal response as subjected to social desirability bias. The main strength of this study that the design was a community-based prospective follow up that gave a relatively good measure of the effect of advanced maternal age on adverse perinatal outcomes. Standard and validated tools used to measure the pregnancy status, baseline assessment to maintain validity and reliability.

In summary, this study intended to fill a research gap in Ethiopia that shows the association of advanced maternal age on perinatal outcomes after controlling possible confounders. Stillbirth and neonatal mortality were significantly associated with advanced maternal age. Nevertheless, pre-term, post-term delivery, and estimated birth weight did not show a significate association. Even if the study design was prospective, the readers should consider the limitations of these other methodological aspects while interpreting the finding, and the other scholars would do more to overcome those limitations. The study will input for policymakers and different stakeholders to design appropriate strategies and planning for interventions.

## Conclusions

This study showed that a significant number of women became pregnant during advanced maternal age. Those adverse perinatal outcomes are unpredictable and unpreventable in the majority of bases. But, highly increased as women age advances. This study identified that stillbirth and neonatal mortality was significantly associated with advanced maternal age. As such, different intervention programs should design to create awareness and to provide counseling services for women with advanced age or delayed childbearing.

## Supplementary information

**Additional file 1.** Tools.

## Data Availability

The datasets generated and/or analyzed during the current study are not publicly available due to anonymity issue but are available from the corresponding author on reasonable request.

## Abbreviations

AM-HDSS: Arba Minch-Health and Demographic Surveillance System sites
BMI: Body Mass Index
HFIAS: Household Food Insecurity Access Scale
ODK: Open Data Kit
