# Supplementary material for: Association between maternal age and adverse perinatal outcomes in Arba Minch zuria, and Gacho Baba district, southern Ethiopia: a prospective cohort study
Source: BMC Pregnancy Childbirth. 2020 Oct 6;20:590. doi: 10.1186/s12884-020-03285-0 (PMC7541292; doi:10.1186/s12884-020-03285-0)
Supplement: Supplementary file 1 — Additional file 1. Tools. [file 12884_2020_3285_MOESM1_ESM.pdf]

Part I: Pregnancy screening checklist

Some background information's and identifications of eligible women

Background Information

1. Kebele: ______________
2. Got: ______________
3. Name of head of the household: ________________________
4. House number: ________________
5. Phone number if available:___________________

List of all 15-49 years old women and their pregnancy information

S.No	List of 15-49 years old female	Age in years	Relation to Head of household	Marital status /cohabitation	Current Pregnancy status*	Approx. GA in weeks	Language preference	Pregnant woman's ID.NO (Code)	Remarks
1									
2									
3									
4									

(*Use the attached pregnancy screening criteria)

Pregnancy screening checklist (Adapted from Whiteman *et al.*, 2014).

Ask this pregnancy screening criteria and indicate eligibility for all women 15-49 years, who assumed to have started sexual experiences.

S.No	Criteria	Responses	Eligibility
**1.**	Are you known pregnant? (Confirmed at health facility or big abdomen with visible pregnancy)	**Yes**	**Eligible**
		**No**	**Go to → 2**
**2.**	Did you have a baby in the last 4 week?	**Yes**	**Non-Eligible**
		**No**	**Go to → 3**
**3.**	Are you exclusively or almost exclusively breastfeeding a baby < 6 months old and have you had no menstrual period since giving birth?	**Yes**	**Non-Eligible**
		**No**	**Go to → 4**
**4.**	Have you had a miscarriage or abortion in the past 7 days?	**Yes**	**Non-Eligible**
		**No**	**Go to → 5**
**5.**	Have you been using a reliable contraceptive method consistently and correctly since last menstrual period or giving birth?	**Yes**	**Non-Eligible**
		**No**	**Go to → 6**
**6.**	Did your last menstrual period start within the past 7 days?	**Yes**	**Non-Eligible**
		**No**	**Go to → 7**
**7.**	Did your last menstrual period start within the past 12 weeks?	**Yes**	**Non-Eligible***
		**No**	**Go to → 8**
**8.**	Have you abstained from sexual intercourse since your last menstrual period or delivery?	**Yes**	**Non-Eligible**
		**No**	**Eligible**

****There may be little chance of having early pregnancy, but not eligible for the study.***

Thanks!

Part II: Baseline information

SNo	Questions	Response	Skip
**Part One: Socio-demographic Characteristics**			
101	Kebele	____________________	
102	Residence	a. Urban<br>b. Rural	
103	Gote	____________________	
104	Name of head of house hold	____________________	
105	Phone number (anyone who are immediate response)	____________________	
106	Health Center in the surround	____________________	
107	Health Post in the surround	____________________	
108	Health extension worker name and phone number	________________________________<br>____________________	
109	How old are you?	___________(in completed year)	
110	What is your ethnicity?	a. Gamo<br>b. Gofa<br>c. Zayse<br>d. Amhara<br>e. Oromo<br>f. Other, specify__________	
111	What is your religion?	a. Orthodox<br>b. Catholic<br>c. Protestant<br>d. Muslim<br>e. Traditional	
112	What is your marital status?	a. Married<br>b. Single<br>c. Widowed<br>d. Divorced<br>e. Separated due to work	
113	What is your educational status of mother?	a. No formal education<br>b. Primary(1-8)<br>c. Secondary(9-12)<br>d. College and above	
114	What is your husband educational status?	a. No formal education<br>b. Primary (1-8)<br>c. Secondary (9-12)<br>d. College and above	
115	What is your occupational status?	a. House wife<br>b. Merchant<br>c. Government employer<br>d. Daily laborer	
116	What is your husband occupational status?	a. Farmer<br>b. Merchant<br>c. Government employer<br>d. Wavier<br>e. Daily laborer	

117	Pre pregnancy weight of the women	___________(in kg)	
118	Height of the women	___________(in meter)	
119	Body mass index	__________(in kg/m2)	
**Part Two: Socio- economic status/ Wealth index questions**			
201	Does any member of this household has own any agricultural land?	a. Yes<br>b. No	
202	Is the land is cultivated?	a. Yes<br>b. No	
203	Does your family have any stored grains/cereals in the house?	a. Yes<br>b. No	
204	Does this household own any livestock, herds, other farm animals, or poultry?	a. Yes<br>b. No	
205	Does your household have electricity?	a. Yes<br>b. No	
206	Does your household have radio?	a. Yes<br>b. No	
207	Does your household have television?	a. Yes<br>b. No	
208	Does your household have mobile or telephone?	a. Yes<br>b. No	
209	Does your household have chair?	a. Yes<br>b. No	
210	Does your household have sofa?	a. Yes<br>b. No	
211	Does your household have table?	a. Yes<br>b. No	
212	Does your household have bed?	a. Yes<br>b. No	
213	Does your household have electric mitad?	a. Yes<br>b. No	
214	Does your drinking water is safer for drink?	a. Yes<br>b. No	
215	Does yours cooking and hand washing water is safe?	a. Yes<br>b. No	
216	Does your household have clean toilet facility?	a. Yes<br>b. No	
217	Does your main materials of floor of the house is made from cement or ceramic tiles?	a. Yes<br>b. No	
218	Does your main materials of roof of the house is made from finished roof (iron, tin, finished wood, cement, ceramic)?	a. Yes<br>b. No	
219	Does your main materials of external of the house is made from finished wall (cement, brick, stone with cement, wood planks)?	a. Yes<br>b. No	
220	Does yours rooms of the house is coinciding or enough to accommodate with number of persons in household?	a. Yes<br>b. No	
221	Do you have a separate room which is used as a kitchen?	a. Yes<br>b. No	
222	Do you use electricity as main source of fuel for cooking?	a. Yes<br>b. No	
223	Does any member of this household have saved money in account?	a. Yes<br>b. No	

224	How much is saved?	______________(ETB)	
225	Do you have any income per month?	a. Yes<br>b. No	
226	In average how much you earned per month?	______________(ETB)	
**Part Three: Household Food Insecurity Related Questions**			
301	In the past four weeks, did you worry that your household would not have enough food?	a. Yes<br>b. No →	303
302	How often did this happen?	a. Rarely (once or twice in the past four weeks)<br>b. Sometimes (three to ten times in the past four weeks)<br>c. Often (more than ten times in the past four weeks)	
303	In the past four weeks, were you or any household member not able to eat the kinds of foods you preferred because of a lack of resources?	a. Yes<br>b. No →	305
304	How often did this happen?	a. Rarely (once or twice in the past four weeks)<br>b. Sometimes (three to ten times in the past four weeks)<br>c. Often (more than ten times in the past four weeks)	
305	In the past four weeks, did you or any household member have to eat a limited variety of foods due to a lack of resources?	a. Yes<br>b. No →	307
306	How often did this happen?	a. Rarely (once or twice in the past four weeks)<br>b. Sometimes (three to ten times in the past four weeks)<br>c. Often (more than ten times in the past four weeks)	
307	In the past four weeks, did you or any household member have to eat some foods that you really did not want to eat because of a lack of resources to obtain other types of food?	a. Yes<br>b. No →	309
308	How often did this happen?	a. Rarely (once or twice in the past four weeks)<br>b. Sometimes (three to ten times in the past four weeks)<br>c. Often (more than ten times in the past four weeks)	
309	In the past four weeks, did you or any household member have to eat a smaller meal than you felt you needed because there was not enough food?	a. Yes<br>b. No →	310
310	How often did this happen?	a. Rarely (once or twice in the past four weeks)<br>b. Sometimes (three to ten times in the past four weeks)<br>c. Often (more than ten times in the past four weeks)	
311	In the past four weeks, did you or any other household member have to eat fewer meals in a day because there was not enough food?	a. Yes<br>b. No →	312

312	How often did this happen?	a. Rarely (once or twice in the past four weeks)<br>b. Sometimes (three to ten times in the past four weeks)<br>c. Often (more than ten times in the past four weeks)	
313	In the past four weeks, was there ever no food to eat of any kind in your household because of lack of resources to get food?	a. Yes<br>b. No →	314
314	How often did this happen?	a. Rarely (once or twice in the past four weeks)<br>b. Sometimes (three to ten times in the past four weeks)<br>c. Often (more than ten times in the past four weeks)	
315	In the past four weeks, did you or any household member go to sleep at night hungry because there was not enough food?	a. Yes<br>b. No →	317
316	How often did this happen?	a. Rarely (once or twice in the past four weeks)<br>b. Sometimes (three to ten times in the past four weeks)<br>c. Often (more than ten times in the past four weeks)	
317	In the past four weeks, did you or any household member go a whole day and night without eating anything because there was not enough food?	a. Yes<br>b. No →	401
318	How often did this happen?	a. Rarely (once or twice in the past four weeks)<br>b. Sometimes (three to ten times in the past four weeks)<br>c. Often (more than ten times in the past four weeks)	
**Part Four: Habit of the mother**			
401	Do you have any habit of smoking cigarette/tobacco?	a. Yes<br>b. No	
402	Do you have any habit of drinking alcohol/local alcohol containing beverage?	a. Yes<br>b. No	
403	Do you use hashish/shisha?	a. Yes<br>b. No	
404	Do you consume local herbs?	a. Yes<br>b. No	

Part III: Follow up survey tool

SNo	Questions	Response	Skip
**Part Five: Basic information's**			
501	Kebele	____________________	
502	Residence	a. Urban<br>b. Rural	
503	Gote	____________________	
504	Name of head of house hold	____________________	
505	Name of neonate	____________________	
506	Age of the neonate	____________(in days)	
507	How long it will take to reach health post from your residential home?	On foot ______minutes<br>By car _______ munities (if any)	
508	How long it will take to reach Health center from your residential home?	On foot ______minutes<br>By car _______ munities (if any)	
509	How long it will take to reach hospital from your residential home?	On foot ______minutes<br>By car _______ munities (if any)	
**Part Six: Maternal and child health service**			
601	Did you see anyone for Antenatal care during the pregnancy of this infant?	a. Yes<br>b. No →	608
602	If, Yes, how many visits?	__________________	
603	Parity	__________________	
604	Where was the place of delivery of this Infant?	a. Hospital<br>b. Health center<br>c. Health Post<br>d. Home of TBA/TTBA<br>e. Home of the respondent<br>f. other (Specify) __________	
605	Mode of delivery	a. Spontaneous vaginally<br>b. Cesarean section<br>c. Assisted delivery	
606	Do you have immediate postnatal care	a. Yes<br>b. No	
**Part Seven: Perinatal Characteristics and Outcomes**			
701	Sex of the neonate	a. Male<br>b. Female	
702	What is the approximate GA (duration of pregnancy) in week?	a. Estimated at term<br>b. Estimated pre-term<br>c. Estimated post-term<br>d. Unknown	
703	How do you rate the size of the neonate at the time of birth?	a. Very Small<br>b. Smaller than usual<br>c. About average<br>d. Larger than usual	
704	Intrauterine fetal death	a. Yes<br>b. No	
705	Neonatal trauma	a. Yes<br>b. No	
706	Does you pregnancy is end up with still birth?	a. Yes<br>b. No →	708

707	If, Yes, what is the possible cause	______________________	
708	Apgar at1 and 5min	___________/__________	
709	Does the neonatal encounter any complications?	a. Yes<br>b. No →	711
710	If the above response is yes	specify_____________________	
711	Baby referred to another facility?	a. Yes<br>b. No	
712	Admitted to special care or intensive care unit?	a. Yes<br>b. No	
713	Does the newborn have any structural anomalies?	a. Yes<br>b. No	
714	Vital status of newborn	a. Alive<br>b. Dead within 7 days<br>c. Dead after 7 days but less than 28 days<br>d. Dead (timing not specified)<br>e. Unknown	

Thanks!
